# Periodontal Regenerative Treatment of Intrabony Defects Associated with Palatal Grooves: A Report of Two Cases

**DOI:** 10.1155/2019/8093192

**Published:** 2019-06-03

**Authors:** Stefano Corbella, Alice Alberti, Beatrice Zotti, Luca Francetti

**Affiliations:** ^1^Department of Biomedical, Surgical and Dental Sciences, Università degli Studi di Milano, Milan, Italy; ^2^IRCCS Istituto Ortopedico Galeazzi, Milan, Italy; ^3^Institute of Dentistry, I.M. Sechenov First Moscow State Medical University, Moscow, Russia

## Abstract

A palatal radicular groove (PRG) is a morphological deformity, occurring during tooth development. It is usually located on the palatal aspect of maxillary incisors and frequently associated with periodontal or endodontic-periodontal lesions. Some treatment options were described for such lesions, including primary endodontic treatment and periodontal surgery and extraction with intentional replantation after removal of a PRG and endodontic treatment. The present paper reported two cases of PRG-associated deep intrabony defects, successfully treated with periodontal surgery with enamel matrix derivative (EMD) application and mechanical removal of PRGs, avoiding endodontic treatment or retreatment. The complexity of the diagnostic process was also discussed.

## 1. Introduction

In scientific literature, palatal radicular grooves (PRGs) were first described by Lee and coworkers in 1968 [1], who discussed its clinical characteristics and potential etiology [1]. A PRG is usually described as a kind of morphological deformity, occurring during tooth development as a failed attempt to form a new root [2], usually located on the palatal aspect of permanent maxillary lateral incisors [3, 4]. From the anatomical point of view, PRGs are heterogeneous, generally extending from the central fossa of the incisor, over the cingulum and down apically, on the root surface [4, 5]. In some cases, the presence of one PRG was considered as an important adverse prognostic factor, because of the frequently associated wide endodontic-periodontal lesions [6]. Even though a recent classification scheme for tooth, root, and canal anomalies was recently proposed [7], the most common classification for a PRG was proposed by Gu [8], who described three different types on the basis of its anatomical extension.

Considering that, in the past, many terms were used to describe this particular kind of deformity (such as palate-gingival groove, development of radicular anatomy, distolingual groove, palatal-radicular groove, and radicular groove [5]), PRG incidence was described, with this limitation, to range between 2.8% and 18% [5, 9–11] and one study on a cohort of Taiwanese people found PRGs in more than 40% of the subjects [12].

With regard to the clinical features, the presence of a PRG provides an ideal pathway for oral bacteria to reach deep periodontal tissues, since it can be difficultly cleaned by standard oral hygiene maneuvers. The apical migration of bacteria could lead to the formation of intrabony pockets, which are located in correspondence of the PRG and are usually deep and narrow [10, 13, 14]. In some cases, probably due to rapid progression of the infection, the lesion could reach the apical tissues, thus causing pulp necrosis and consequently an endodontic-periodontal lesion of periodontal origin [2, 9, 11, 15, 16]. In some cases, the diagnosis could be complicated by the presence of the sinus tract usually on the vestibular mucosa, in correspondence of the affected tooth [4].

The treatment options for endodontic-periodontal lesions related to the presence of a PRG include primary endodontic treatment [4] usually associated with periodontal surgery that was limited in the site of the defect [5, 9, 15]. Other authors described the extraction of the affected tooth when a complete loss of the periodontal support occurred [4] or extraction and intentional replantation after removal of a PRG and extraoral endodontic treatment [11]. Ideally, a timely diagnosis could prevent the pulpal involvement, thus allowing successful treatment of the defect through a merely periodontal approach.

The aim of this paper was to present two case reports of PRG-associated deep intrabony defects treated with periodontal surgery and enamel matrix derivative (EMD) application, avoiding endodontic treatment or retreatment.

## 2. Case Presentation

Both subjects were treated following the principles of the Helsinki Declaration [17]. The operator (SC) informed the patients about the treatment alternatives and about the planned intervention, and both patients signed a written informed consent form before performing the treatment procedures.

### 2.1. Case Report 1

One female patient, aged 49 at the time of the first visit, without any systemic disease (ASA-1 following the classification proposed by the American Society of Anesthesiologists) presented referring an episode of acute abscess in the region of 1.1, 1.2, and 1.3 that was treated with systemic administration of Amoxicillin 875 mg+Clavulanate 125 mg twice a day for six days.

**Figure 1:**
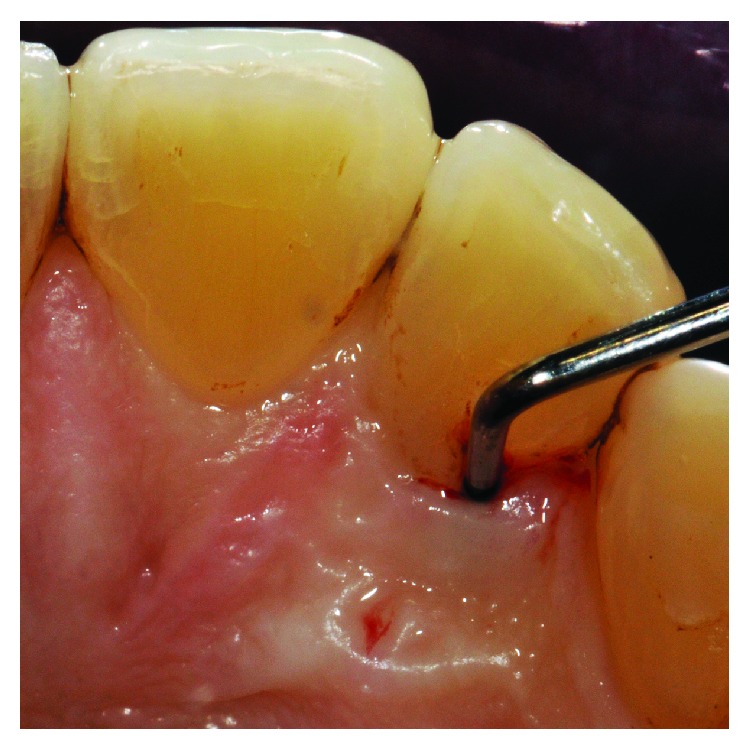
Clinical examination revealed the presence of a 13 mm deep periodontal pocket associated with an enamel alteration on the palatal side of 1.2.

**Figure 2:**
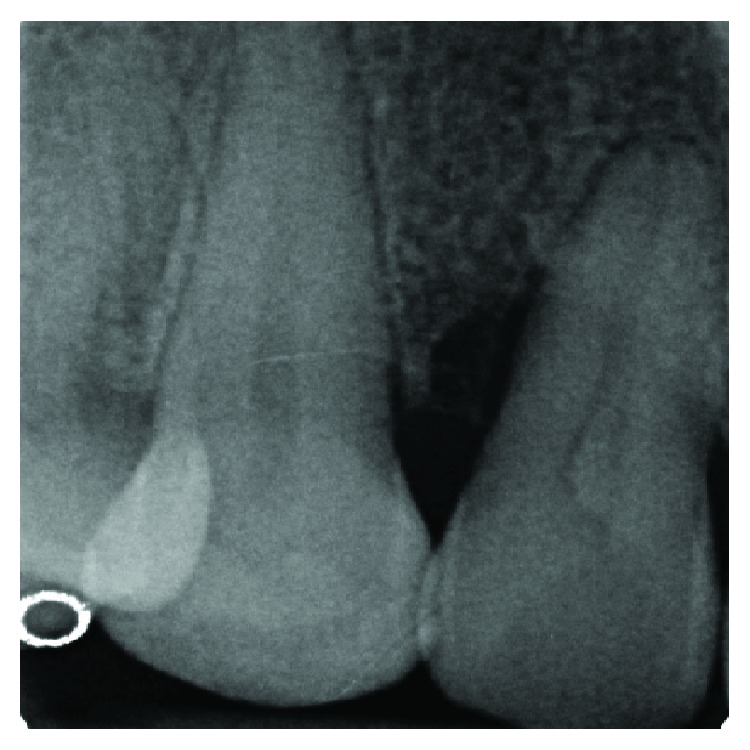
An angular bone defect was also visible through intraoral radiographic examination.

The clinical examination revealed the presence of one isolated periodontal pocket (measuring 13 mm) in correspondence of the palatal aspect of 1.2, in the presence of one enamel alteration (PRG) (Figure 1). Mild pain was associated with percussion of 1.2, and the element was vital, without any caries. Full-mouth clinical evaluation excluded the presence of generalized periodontitis. Adjacent sites did not show the presence of any pathological periodontal pocket, with probing depth less than 4 mm in all sites. Radiographic examination showed a deep intrabony defect distal to 1.2 (Figure 2).

Differential diagnosis reasonably excluded the presence of a vertical root fracture (VRF) since the tooth was vital and no history of trauma was reported. The treatment option was to elevate a palatal flap to allow the debridement of the pocket, to remove physically the etiologic factor (the PRG) maintaining tooth vitality, and to stimulate periodontal regeneration by using enamel matrix derivative (EMD).

**Figure 3:**
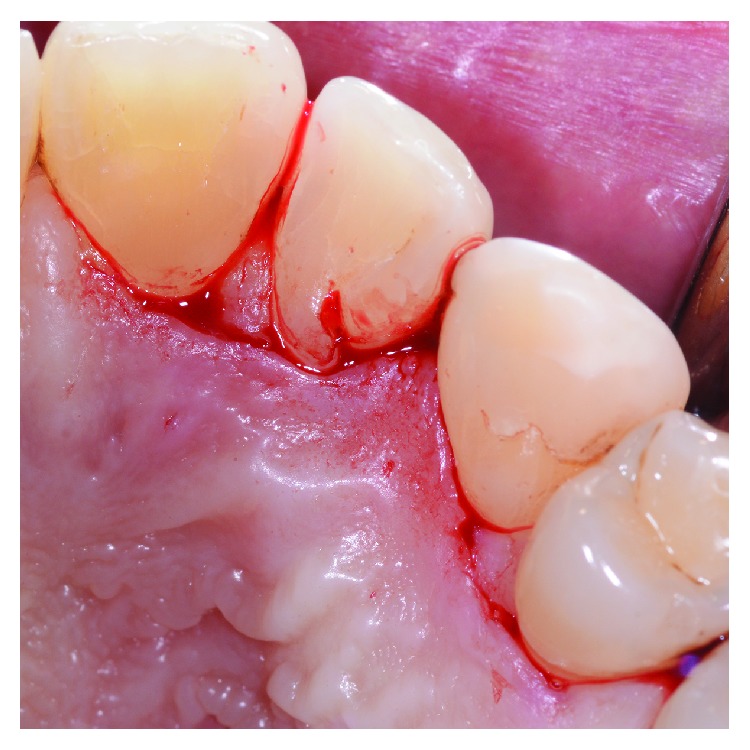
A horizontal incision on the palatal aspect was performed, preserving the interdental papilla.

**Figure 4:**
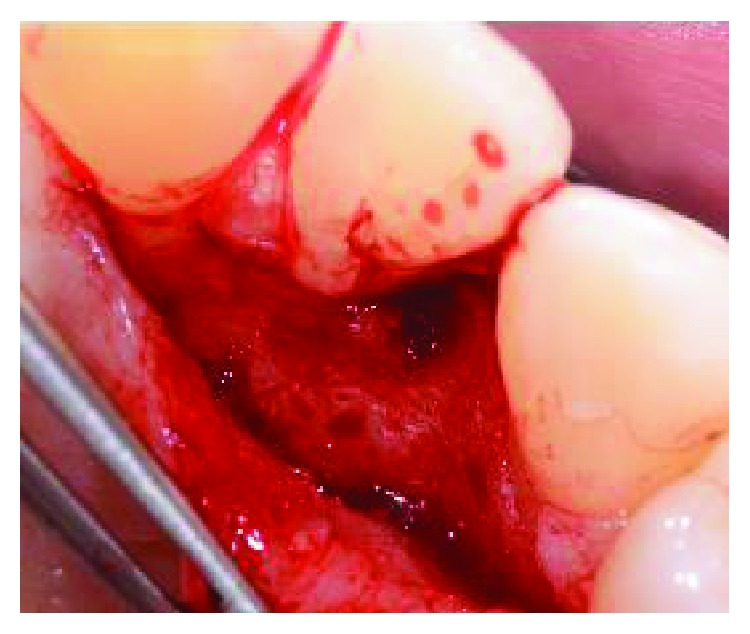
The reflection of the flap, including the papilla between 1.2 and 1.3, allowed a direct visualization of the intrabony defect and the radicular groove.

**Figure 5:**
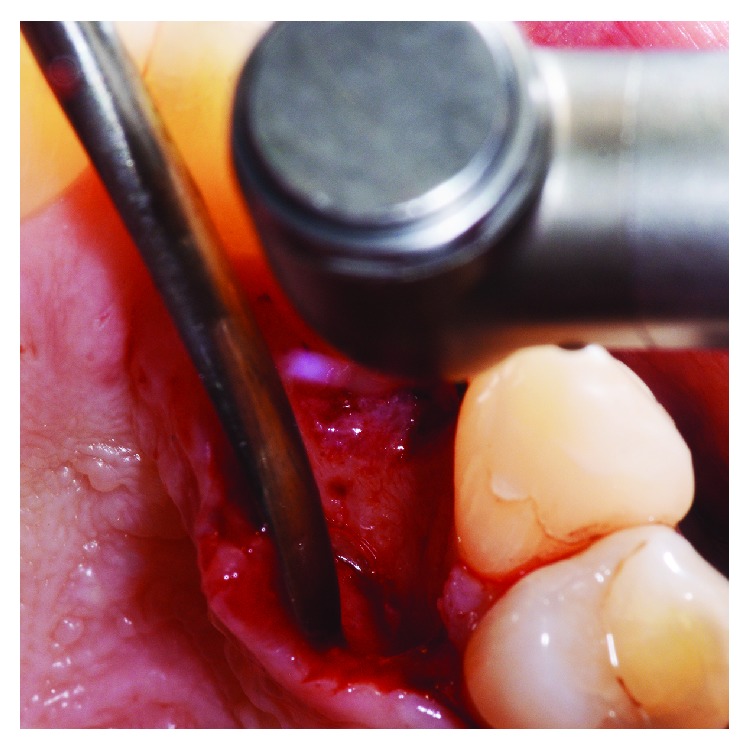
Granulation tissue was removed from the intrabony defect.

**Figure 6:**
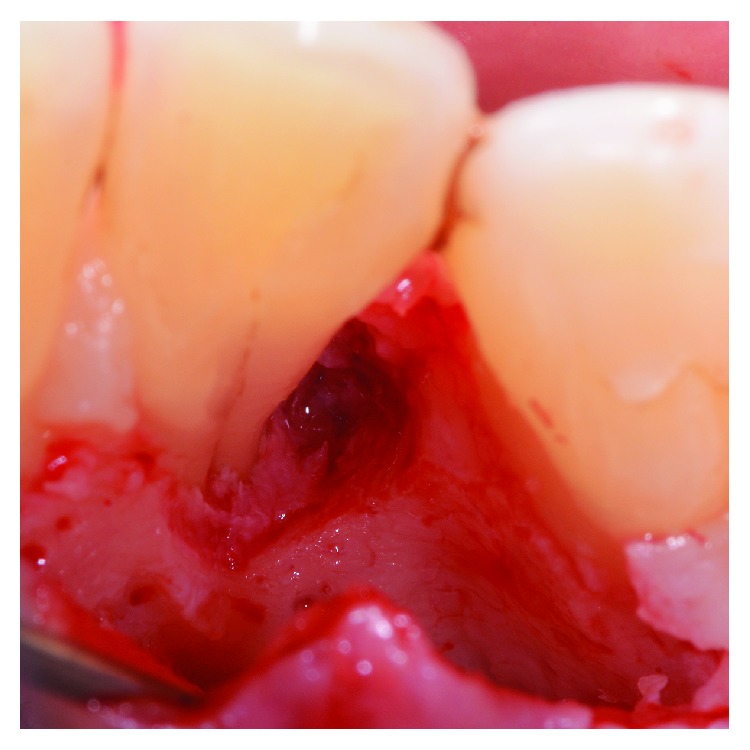
Using a diamond bur with copious irrigation of sterile saline solution, the enamel alteration was removed.

**Figure 7:**
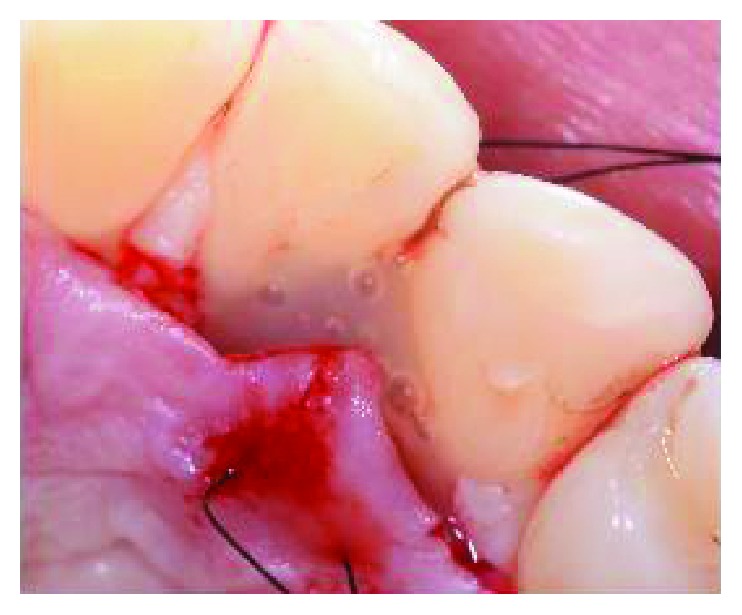
The bone substitute was placed in the osseous defect after it had been conditioned through a 24% EDTA gel and irrigated with abundant sterile saline solution.

**Figure 8:**
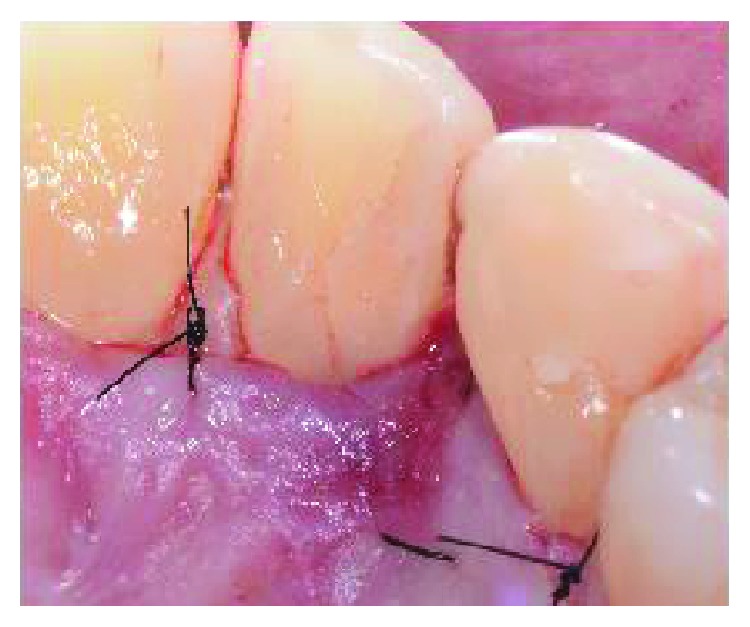
Single 5/0 and 6/0 sutures were placed.

After local anesthesia on the vestibular and palatal side with Articaine 4%+epinephrine 1 : 100.000, a horizontal incision was performed on the palatal aspect preserving interdental papilla between 1.1 and 1.2 and between 1.3 and 1.4 (Figure 3). The papilla between 1.2 and 1.3 was separated from the vestibular portion and reflected in order to directly visualize the PRG and the defect (Figure 4). After the debridement of the defect, by removing the granulation tissue, the PRG was smoothed by the use of a diamond bur under abundant irrigation of sterile saline solution (Figures 5 and 6). Following the instructions provided by the manufacturer, tooth surface was conditioned for two minutes with 24% EDTA gel (Straumann® PrefGel®, Straumann AG, Basel, Switzerland) and, after its removal through copious irrigation with sterile saline solution, EMD (Straumann® Emdogain®, Straumann AG, Basel, Switzerland) was placed in the defect (Figure 7). The flap was then sutured with single 5/0 and 6/0 sutures (ETHILON®, Ethicon Inc., Johnson & Johnson, Piscataway, NJ, United States) (Figure 8). The patient was advised to avoid any trauma or traction in the region of surgery and not to consume hard food during the first five days. Ibuprofen 400 mg was prescribed twice a day for three days for inflammation control and pain relief. Healing completed without any adverse event.

**Figure 9:**
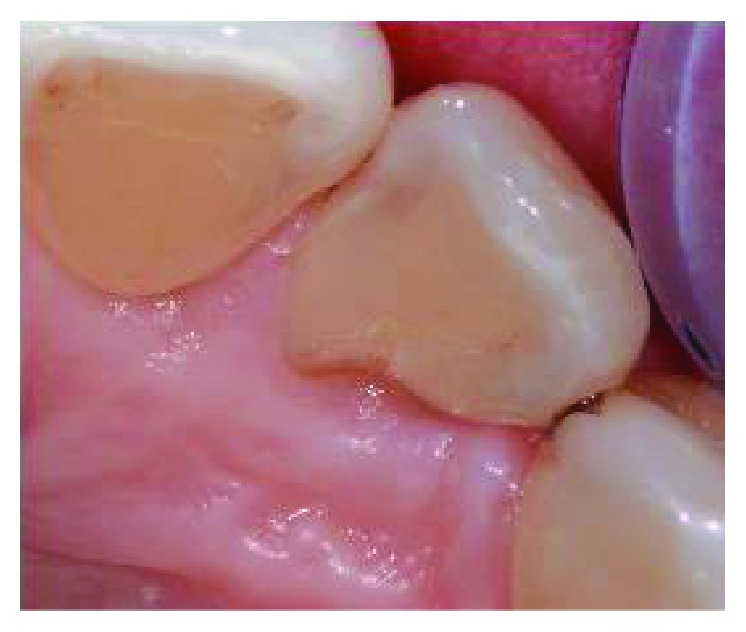
Clinical examination after 12 months.

**Figure 10:**
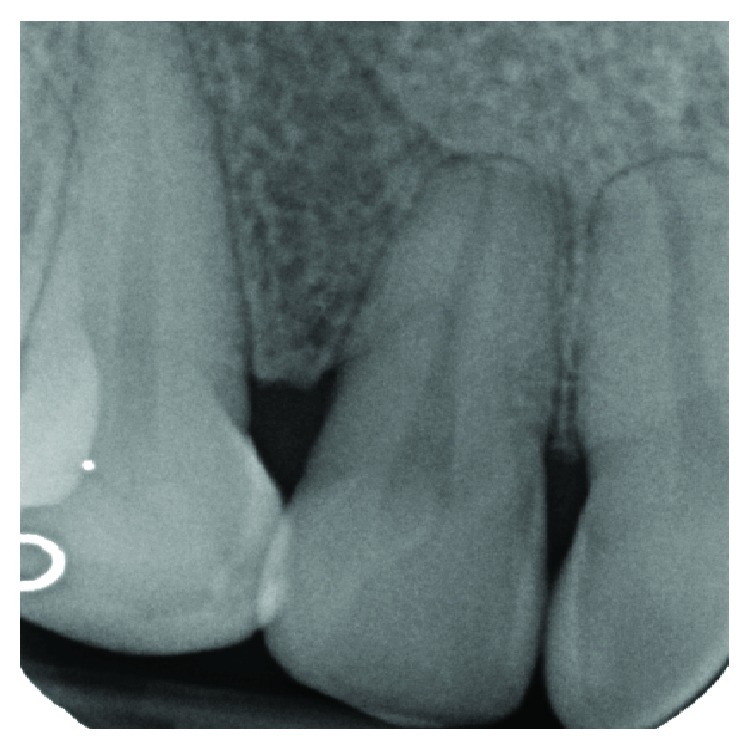
Radiographic examination after 12 months.

Clinical and radiographic examination 12 months after surgical intervention revealed a complete healing with bone regeneration in the site of the defect (Figures 9 and 10).

### 2.2. Case Report 2

One female patient, aged 36 at the time of the first visit, without any systemic disease (ASA-1 following the classification proposed by the American Society of Anesthesiologists) presented referring mild pain sensation while touching with the tongue the palatal-gingival margin of 1.2.

**Figure 11:**
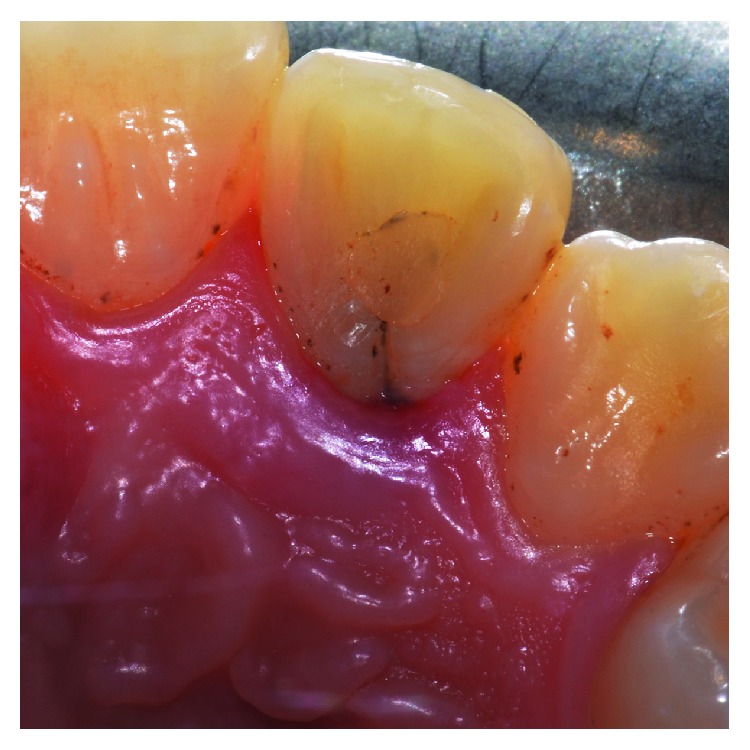
Clinical examination: a 10 mm periodontal pocket is associated with 1.2 in correspondence of a palatal groove.

**Figure 12:**
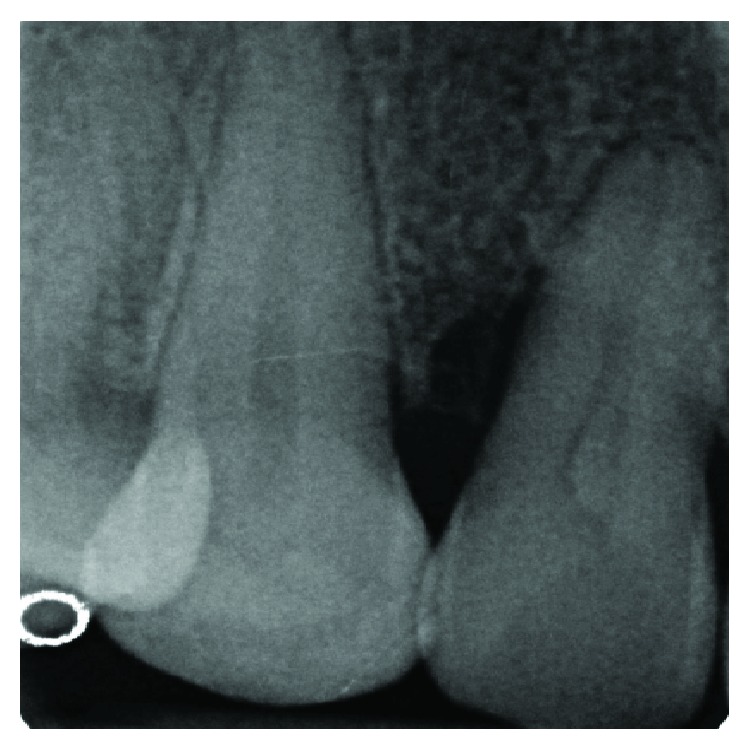
Radiographic examination: no bone resorption is visible in correspondence of the involved tooth.

The clinical examination revealed the presence of one deep narrow isolated periodontal pocket (PD = 10 mm) in correspondence of the palatal aspect of 1.2, in the presence of bleeding on probing, no plaque accumulation, and one PRG (Figure 11). Full-mouth clinical evaluation excluded the presence of periodontitis. Radiographic examination showed no evidence of one visible intrabony defect (Figure 12). The tooth was endodontically treated and did not show signs and symptoms of a lesion of endodontic origin.

**Figure 13:**
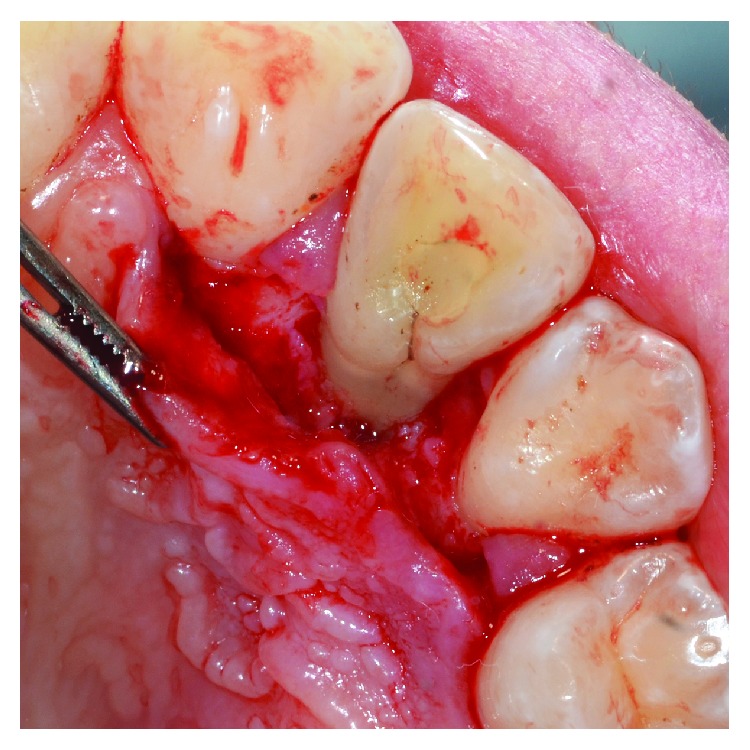
After a flap was reflected and direct visualization of the defect was obtained, debridement of the periodontal lesion was performed and the radicular groove was smoothed.

**Figure 14:**
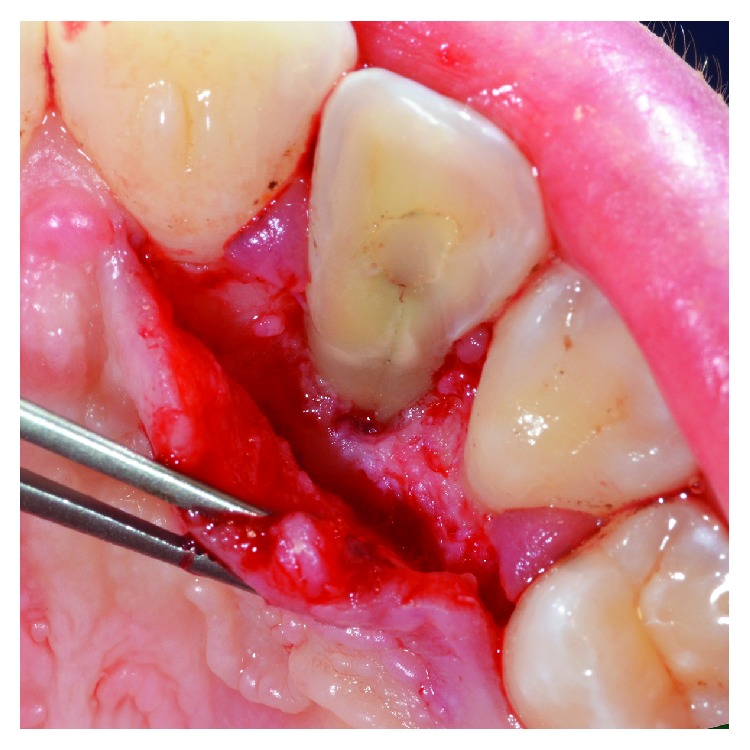
The radicular groove was smoothed using a diamond bur.

The treatment was by a regenerative approach, using EMD, on the palatal aspect, removing mechanically the PRG from the root (Figures 13 and 14), following the technique described in detail before.

**Figure 15:**
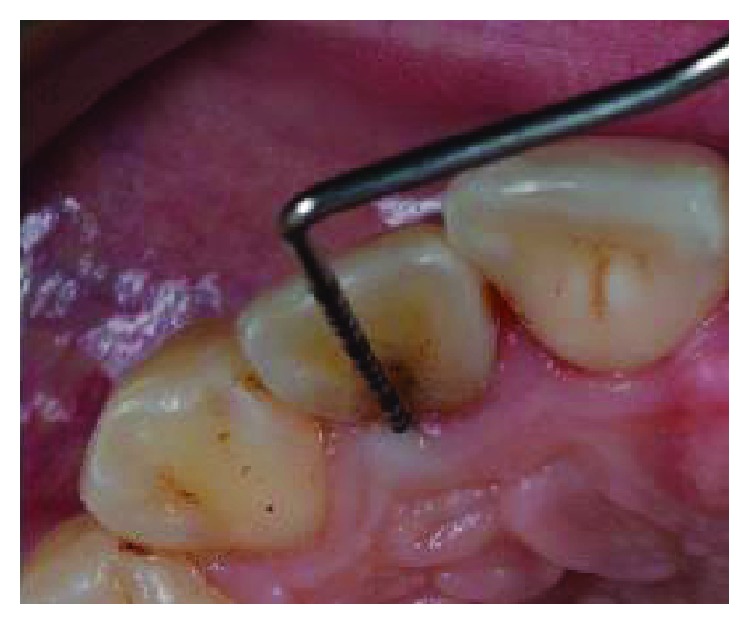
Clinical examination after 12 months.

**Figure 16:**
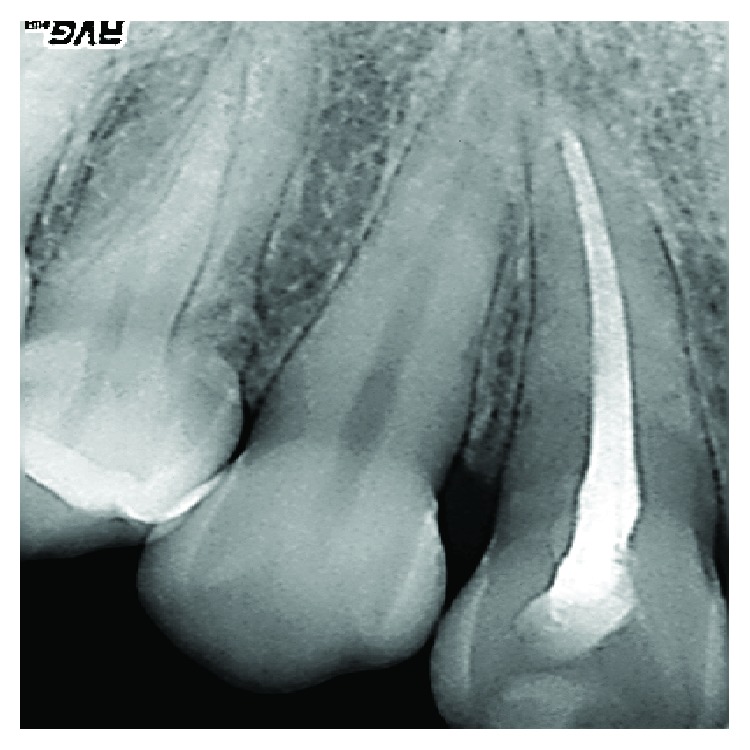
Radiographic examination after 12 months.

After one year from surgical intervention, clinical examination showed a physiological probing depth (less than 4 mm) in the site of the surgery and the absence of symptoms (Figures 15 and 16).

## 3. Discussion

The described case reports showed the clinical and radiographic success of one treatment option for PRG-related intrabony lesions. The periodontal regenerative treatment resulted in the resolution of the cases, maintaining tooth vitality when present and avoiding the recurrence of symptoms.

The presented results should be considered with caution, in particular when evaluating external validity, since they are derived on just two cases not included in one clinical trial. Even considering this important limitation, some considerations could be made on the basis of the outcomes obtained.

The first issue to be discussed is about the diagnostic procedure that led to the diagnosis of PRG-related intrabony defect.

Both patients referred some nonspecific symptoms that could be related to various conditions of periodontal or endodontic origin. In order to identify the etiologic factor, an accurate clinical examination was performed, revealing no caries, no restorations, and the presence of a deep and narrow periodontal pocket on the lingual aspect of the tooth.

Purely periodontal lesions could be obviously related to the presence of periodontal disease. In the presented cases, a full-mouth examination excluded the presence of periodontitis. For this reason, we considered other factors which can be related to localized periodontal lesions, with or without acute abscess. Those include endodontic periapical or lateral lesions of the involved or of an adjacent tooth which find a drainage route through the periodontal ligament and the gingival sulcus [18], vertical root fractures with or without pulpal involvement, local plaque retention factors, such as the presence of inadequate restorations [19], developmental anomalies such as gemination, fused roots, and radicular grooves [1], and other rare conditions, such as ectopic gingival sebaceous glands [20]. All these options should be considered and tested.

Even though no caries was visible, no previous restoration was present, and no dental trauma was reported by the patients, the hypothesis of a purely endodontic lesion was not excluded since it can also result, for example, from longitudinal tooth fractures such as split tooth or VRF, which cannot be easily detected [21]. Thus, tooth vitality was tested and a radiographic examination was performed; in both cases involved, the presence of a fracture was excluded.

Vertical root fractures are usually related to dental trauma and excessive occlusal forces, and they are referred to as “acute” and “chronic” or “fatigue fractures,” respectively [22]. They usually occur in restored endodontically treated teeth with or without endodontic posts, even though they can be encountered in vital and nonendodontically treated teeth [23–25]. The diagnosis of VRFs can be challenging, since signs and symptoms are not specific, including pain with lateral percussion or while chewing, gingival swelling, sinus tract, and periodontal defect. Only the presence of two deep and narrow periodontal pockets on the buccal and lingual sides of the tooth can be considered a pathognomonic sign of VRF. Its radiographical features are also heterogeneous, varying from vertical bone resorption to radiographic thickening of the periodontal ligament and to the typical “halo” effect, a combined periapical and perilateral radiolucency [22, 26]. Surgical exploration, consisting of direct visualization after lifting a full-thickness flap, can confirm VRF diagnosis. In the reported cases, no characteristic sign of VRF was found and no previous endodontic treatment or large restoration was present; on the other side, PRGs typically occur in maxillary lateral incisors [3, 4].

Once the diagnosis of a PRG is confirmed (through direct visualization in the presented cases), some authors proposed a classification of such deformity, distinguishing three types of grooves on the basis of their depth evaluated before intervention by Cone-Beam Computed Tomography (CBCT) [4]. Other classifications were proposed on the basis of groove characteristics as evaluated after tooth extraction [8].

With regard to the treatment options, a combined approach (both from the endodontic side and the periodontal side) was widely described and proposed when the bony defect caused the loss of tooth vitality [5, 9, 11, 15, 16]. Schwartz and coworkers in 2006 described a combined endoperiodontal treatment that was complicated by the complex root canal anatomy [5]. Other authors proposed the extraction, endodontic treatment, and sealing of the groove using composite resin or glass ionomer cement and subsequent replantation of the affected teeth [11, 27]. Al-Hezaimi and coworkers associated intentional extraction and replantation with EMD application to treat residual bone defect [28]. Cho and coworkers published in 2017 the description of three cases treated with a combined endoperiodontal approach with odontoplasty in order to remove/flatten, after endodontic treatment, the groove from root surface [16].

Some studies reported the results of EMD application alone for the treatment of periodontal defects associated with a PRG, in teeth with preserved pulp vitality [29, 30], even though the techniques described were different from the one adopted for the described case reports. However, the use of such biomaterial found a wide justification in previously published scientific papers on the regenerative treatment of deep and narrow periodontal defects, mainly self-contained [31].

Many reviews described the mechanisms of action of EMD in the stimulation of a number of cells (fibroblast and epithelial cells of the gingiva and of the periodontium) involved in the regenerative process, by influencing cell attachment, spreading, proliferation, and, finally, bone remodeling [32–34].

With regard to the clinical applications of EMD, since the first paper published by Heijl in 1997, the results of a number of studies confirmed the efficacy of EMD (even when used alone) in the treatment of deep intrabony defects [35, 36].

The multicentric study by Tonetti and coworkers, published in 2002, compared open flap debridement and EMD application alone in the treatment of intrabony defects [35]. The authors found that the use of EMD resulted in a mean PD reduction of 3.9 ± 1.7 mm and CAL gain of 3.1 ± 1.5 mm 12 months after surgery. These results were similar to those obtained in other studies comparing the same treatment options [36, 37]. Our case reports confirmed that EMD alone could be successfully used for the treatment of PRG-related periodontal defects, when an adequate primary closure was obtained.

In conclusion, the two case reports described a technique that resulted in clinical and radiographic resolution of PRG-related periodontal defect by a periodontal regenerative approach with the application of EMD, furthermore preventing loss of tooth vitality (Case 1). Well-designed randomized controlled clinical trials with an adequate sample size are needed in order to establish which treatment procedure would lead to better results. However, even considering the limitations of the present study, we can speculate that a timing and early diagnosis of PRG-related defect can modify importantly the prognosis of the affected teeth.
